# The role of ion exchange in the passivation of In(Zn)P nanocrystals with ZnS

**DOI:** 10.1038/srep22818

**Published:** 2016-03-14

**Authors:** Deok-Yong Cho, Lifei Xi, Chris Boothroyd, Beata Kardynal, Yeng Ming Lam

**Affiliations:** 1 IPIT and Department of Physics, Chonbuk National University, Jeonju 54896, Republic of Korea; 2 Institute of Solar Fuels (EE-IF), Helmholtz Zentrum Berlin (HZB), 12489 Berlin, Germany; 3 Ernst Ruska Centre (ER-C), PGI-5, Forschungszentrum Jülich, 52425 Jülich, Germany; 4 Semiconductor Nanoelectronics (PGI-9), Forschungszentrum Jülich, 52425 Jülich, Germany; 5 School of Materials Science and Engineering, Nanyang Technological University, 639798, Singapore

## Abstract

We have investigated the chemical state of In(Zn)P/ZnS core/shell nanocrystals (NCs) for color conversion applications using hard X-ray absorption spectroscopy (XAS) and photoluminescence excitation (PLE). Analyses of the edge energies as well as the X-ray absorption fine structure (XAFS) reveal that the Zn^2+^ ions from ZnS remain in the shell while the S^2−^ ions penetrate into the core at an early stage of the ZnS deposition. It is further demonstrated that for short growth times, the ZnS shell coverage on the core was incomplete, whereas the coverage improved gradually as the shell deposition time increased. Together with evidence from PLE spectra, where there is a strong indication of the presence of P vacancies, this suggests that the core-shell interface in the In(Zn)P/ZnS NCs are subject to substantial atomic exchanges and detailed models for the shell structure beyond simple layer coverage are needed. This substantial atomic exchange is very likely to be the reason for the improved photoluminescence behavior of the core-shell particles compare to In(Zn)P-only NCs as S can passivate the NCs surfaces.

Semiconductor nanocrystals (NCs) provide a range of useful properties that are unique to this group of materials. One such commonly encountered NC is III–V indium phosphide (InP) NCs. Their size-tunable properties such as absorption and photoluminescence wavelengths and low intrinsic toxicity compared to II–VI compounds, such as cadmium selenide, cadmium telluride, etc., make these NCs highly attractive for optoelectronic applications^1^,^2^,^3^,^4^. InP in its bulk, thin film or NC form has been used in applications such as light emitting diodes, biomedical labeling, solar cells, sensors, etc^4^,^5^.

Although InP NCs have good theoretical properties inherent to this material, they suffer from non-radiative recombination effects due the presence of surface traps and defects^5^,^6^,^7^. As their sizes are in the nanometer regime, this situation is further aggravated by the large surface contribution. Hence in order to reduce these recombination effects, InP is commonly coated with a zinc sulfide (ZnS) shell. One reason for the choice of ZnS as the shell material, other than ZnS being non-toxic, is that it is a wide bandgap (~3.5 eV) material^8^ and is able to confine the charge to within the cores so that radiative recombination occurs within the core^9^. This shell can also act as a physical barrier between the core and the surrounding medium rendering the NCs more stable to changes in the environment, more resistant to photo-oxidative degradation or changes in the surface chemistry. In fact, in the recent work by Xi *et al.*, ZnS single molecular precursor complexes were decomposed during synthesis to give off hydrogen sulfide gas which results in etching of the InP surface, hence reducing the defects on the surface of these NCs^10^.

Though many advantages can be associated with a ZnS shell on InP NCs, little is known about the local structure of the shell atoms because the typical thickness of the shell is less than a few nm, too thin to possess a sufficiently long-ranged structural order to be measurable by X-ray diffraction. Moreover, the chemical states of the NCs cannot be analyzed reliably with conventional chemical spectroscopic techniques such as X-ray photoelectron spectroscopy (XPS) or Auger electron spectroscopy because their probing depths are typically <5 nm, too small to observe the signals from the core atoms buried under the shell for larger crystals, and more importantly, they are strongly subject to surface contamination during the preparation of the specimens.

In this contribution, we instead utilized hard X-ray absorption spectroscopy (XAS) to simultaneously examine the chemical states and the local structure for each atomic species in the NCs. The chemistry or valence of each atomic species can be identified by the energy of the absorption edge, while the local structure near the ions can be examined by analyzing the oscillatory features far above the edge, namely, the x-ray absorption fine structures (XAFS)^11^. Furthermore, the probing depth for hard XAS is typically a few hundreds of nm, far exceeding the diameter of the NCs, so that the peak intensity in the XAS spectrum is directly proportional to the number of atoms irrespective of their positions. By inspecting the evolution of features in the XAS spectrum for each atomic species with increasing shell growth time, we have determined the atomic exchanges at the core-shell interfaces. This is the first time that such in-depth analysis of the local structure and chemical states of core-shell nanoparticles has been carried out and this demonstrates the possibility of using this technique to gain more understanding of what happens at the interface between the core and shell of nanostructures and also the shell growth process.

## Results and Discussion

**Figure 1:**
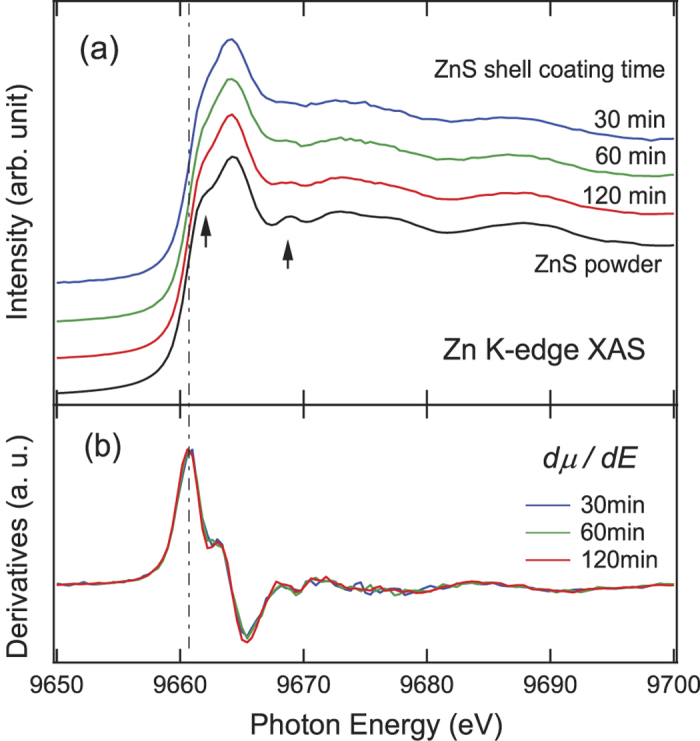
(**a**) Zn K-edge XAS spectra and (**b**) their derivatives from In(Zn)P/ZnS NCs as a function of increasing ZnS coating time. A spectrum from commercial bulk ZnS powder is also shown for comparison. The dashed line indicates the edge energy determined by the first maxima in the derivative spectra.

Figure 1 shows (a) Zn K-edge XAS spectra (*μ*(*E*)) and (b) their derivatives (*dμ*(*E*)/*dE*) from In(Zn)P/ZnS NCs as a function of increasing ZnS coating time. The spectra were normalized to maintain a constant edge size (the difference between the intensities at energies far above and below the edge). The edge energy was determined as the energy of the first steepest slopes in the XAS spectrum or the maximum in the derivative spectrum, as shown by vertical line in Fig. 1. It can be seen from Fig. 1a,b that the edge energies of the three In(Zn)P/ZnS NC samples were almost identical to that of the ZnS powder (Zn^2+^), indicating identical chemistry.

Furthermore, the XAFS features of the three samples were very similar to those of the ZnS powder (Fig. 1a; highlighted by the arrows), except for a slight broadening of features presumably caused by some structural disorder in the shell. This similarity can be seen more clearly in the derivative spectra (Fig. 1b), indicating negligible difference in the local structure near the Zn atoms (zinc blende). The contribution from the Zn atoms in the In(Zn)P core, inferred from the spectrum of a bare In(Zn)P core, was negligible. The features in the spectrum from the core were much weaker and broader compared to the spectra from the In(Zn)P/ZnS samples. Therefore, it can be concluded from Fig. 1 that no substantial evolution of the Zn environment was observed with increasing ZnS shell growth time. This suggests that the migration of the Zn atoms from the ZnS shell into the In(Zn)P core was insignificant.

**Figure 2:**
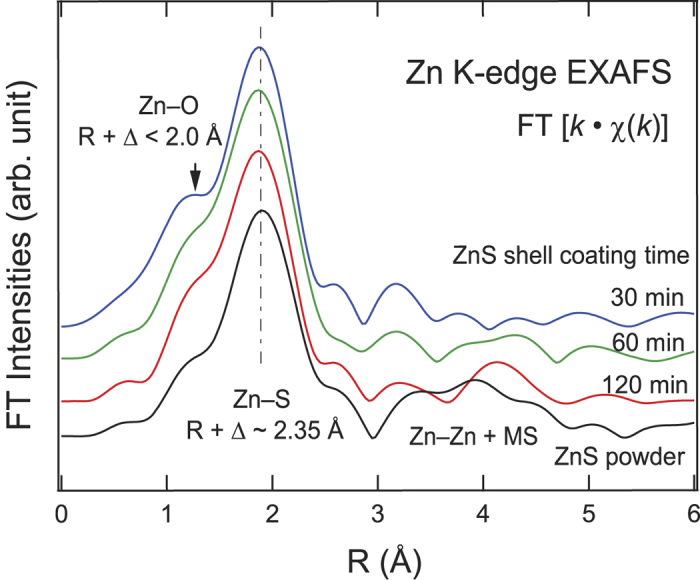
Fourier-transformed (FT) intensities of the *k*^1^-weighted Zn K-edge EXAFS.

Figure 2 shows the Fourier-transformed (FT) intensities of the *k*^1^-weighted Zn K-edge EXAFS taken in a momentum (*k*) range of 3 to 9 Å^−1^. Overall, the features reflect the bonding environment at various distances from the Zn atoms. Thus, the peaks can be assigned to the bonds according to the phase-uncorrected interatomic distance *R*: the main peak near *R* = 1.9 Å should be attributed to Zn-S bonds, while the smaller peak near *R* = 1.3 Å corresponds to Zn bonds to ionic species with a smaller atomic size than for S, and in this case, this is most likely to be O. The peak at 1.3 Å is superimposed on a ripple which is an artifact of Fourier transforming data with a limited range. In additional to these peaks, several features originating from indirect bonds, such as Zn-Zn, as well as multiple scattering (MS), are observed in the data from powdered ZnS at *R* >1.9 Å. The absence of such strong features in the data from the nanocrystals implies substantial structural disorder in the ultrathin ZnS shell. Any small peaks that one would expect from disordered systems are masked in the data by the FT ripples that are particularly clear at *R* > 1.9 Å.

The Zn-S bond length estimated after the scattering phase correction (Δ) is *R* + Δ ~ 2.33 Å for all the In(Zn)P/ZnS samples, while the bond length is 2.35 Å for the ZnS powder. It is evident that the overall FT intensities and *R* values for the Zn-S bonds in the In(Zn)P/ZnS samples are largely maintained. The negligible difference in the bond length from the bulk powder suggests the molecular ZnS shell is hardly strained by In(Zn)P core.

On the other hand, the peak near *R* = 1.3 Å is enhanced in the ZnS shell sample coated for 30 min when compared to ZnS powder. Although it overlaps with the ripple from the FT, its enhancement can be attributed to the formation of Zn-O bonds. At the early stage of shell growth, the oxide layer at the In(Zn)P NC surfaces^10^ would not be eliminated completely by the etching process. The unstable oxide layers are likely to accept Zn atoms to form a small amount of Zn-O bonds at the surface. However, as the shell coverage progresses, such oxide layers are reduced such that Zn-O bonding can be hardly detected. This is consistent with the reduced intensity of the peak as the shell coating time increases (Fig. 2).

**Figure 3:**
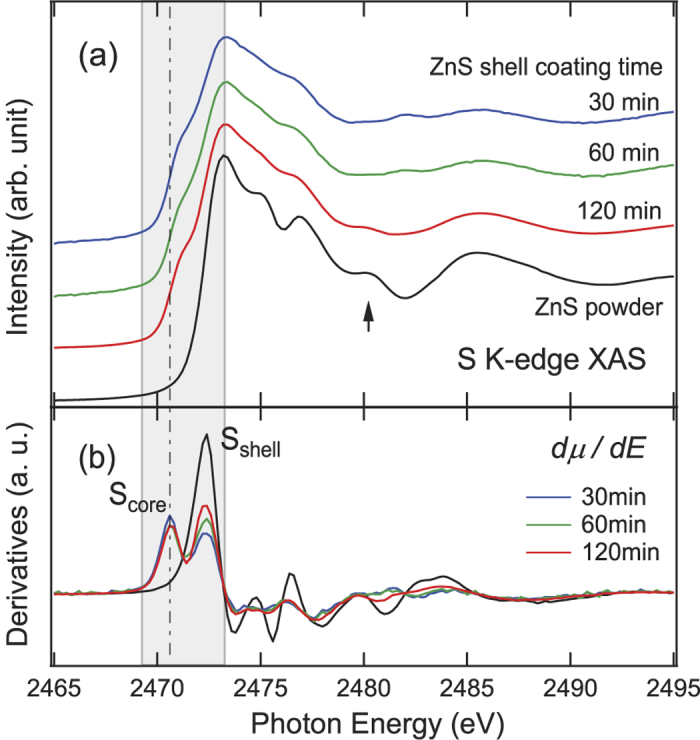
(**a**) S K-edge XAS spectra and (**b**) their derivatives from In(Zn)P/ZnS NCs as a function of ZnS coating time. A spectrum from commercial bulk ZnS powder is also shown for comparison. The dashed line indicates the edge energy. The contributions from S ions in the In(Zn)P core (S_core_) can be clearly distinguished from those in the shell (S_shell_) as shown in the energy range of the shaded area.

On the other hand, the chemistry of sulfur showed a strong evolution with shell growth time. Figure 3 shows normalized S K-edge (S 1s→3p) XAS spectra from the three core-shell samples as well as the reference ZnS powder. Similar to the XAS from the Zn K-edge, the features near the absorption edge (highlighted by vertical line) reflect the chemistry of the S ions, while the XAFSs (highlighted by the arrow) above the edge reflect the local structure around the S ions. Shoulders at low energy (~2471 eV) are clearly observed in the spectra of the three core-shell samples, while they are absent in the ZnS powder spectrum (Fig. 3a). In contrast to the striking difference in the features near the edge, the XAFS from the NCs gradually changes toward those of the ZnS powder as the reaction progresses. This obviously shows that the shoulder cannot originate from sulfur in the ZnS shell.

The intensity of the shoulder remains roughly constant whereas that of the peak of highest intensity (~2473 eV) increases with increasing ZnS coating time. Such contrast in the evolution of the intensity of the two features is prominent in the derivative spectra (Fig. 3b), and is highlighted in the shaded region of Fig. 3. Because of the nature of the spectral evolution with ZnS growth time and their absence in the case of ZnS powder, we assigned the low energy shoulders to the S^2−^ ions in the core (S_core_). This implies the formation of S-In bonds in the core NCs. The main peaks were assigned to the S^2−^ ions in the shell (S_shell_). The decreasing ratio of the [S_core_]/[S_shell_] intensity with increasing relative ZnS thickness is reasonable in that the relative concentration of the core becomes lower as ZnS was added while the core was etched. The low energy shoulder becomes readily more intense even for the NCs with the short ZnS shell growth time (30 min), strongly suggesting that the S penetration into the In(Zn)P core was promoted even at an early stage of the NC synthesis. Therefore, it can be concluded from Fig. 3 that S atoms from the shell do indeed penetrate into the core, whereas Zn atoms do not.

**Figure 4:**
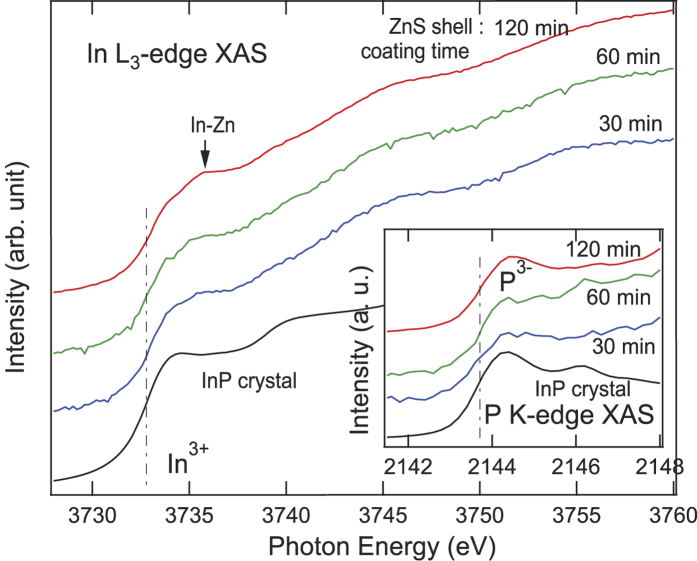
In L_3_-edge XAS spectra with inset P K-edge XAS spectra from In(Zn)P/ZnS NCs as a function of increasing ZnS coating time. A spectrum from a commercial InP crystal is also shown for comparison. The dashed lines indicate the edge energies.

We observed from the XAS at the edges of the shell atoms (Figs 1 and 3) that only S ions in the shell were incorporated into the core. The effect of the anionic incorporation was scrutinized by examining the local chemistry of the core ions (In and P). Figure 4 shows normalized In L_3_-edge (In 2p_3/2_→4d/5s) XAS spectra from the three NCs. A spectrum from a bulk InP crystal is also shown for comparison. As shown by the vertical line, the edge energies were roughly maintained for all NCs, being coincident with that of the InP crystal. This confirms the +3 valence of the In ions in the NCs. Aside from the first main peak at ~3733 eV, a prominent peak at ~3735 eV was observed for all the NCs. The absence of such a peak in the InP crystal spectrum implies that the feature is related to the presence of Zn in the In(Zn)P core. In^3+^ ions surrounded by Zn^2+^ ions would be slightly re-oxidized and thus have a higher peak energy than for InP. Hence, the strong shoulder peak was assigned to In-Zn bonds.

A close inspection of the peak intensity revealed that the In-Zn bond peak at ~3735 eV becomes more pronounced as the ZnS shell growth time increases. This reflects an increase in the number of In-Zn bonds, which appears to be contradictory to the finding from Fig. 1 that Zn atoms from the shell do not penetrate into the core. While the signature of In-Zn bonds is mainly from the In(Zn)P core itself, the increase in the number of the In-Zn bonds should be accounted for by additional bonding at the core-shell interface. If the shell covers the core fully, the number of In-Zn bonds at the core-shell interface should remain constant. Therefore, the increasing number of In-Zn bonds with the addition of ZnS indicates that the shell coverage completed slowly. At an early stage in the shell coating (e.g. after 30 min of coating time), the shells only partly cover the In(Zn)P cores. The number of interfacial In-Zn bonds should increase as the shell coverage increases, finally reaching full coverage after a long ZnS shell growth time (e.g. after 120 min of coating time).

The inset in Fig. 4 shows the P K-edge (P 1s→3p) XAS spectra from the three NCs as well as the InP crystal. Because of the residual phosphine or P-containing organics outside the NCs, the spectra also contained strong features from oxidized phosphorus such as P^3+^ or P^5+ ^12. Nevertheless, we observe that the edges of the lowest energy peaks were roughly maintained (highlighted by vertical line in the inset). This shows that the valence of the P ions in the core remained as −3 for all the samples.

**Figure 5:**
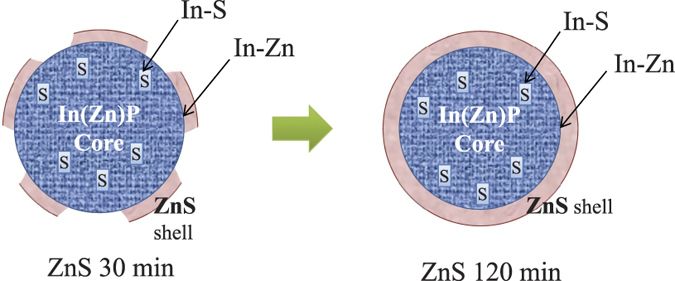
Schematic diagram showing the core-shell atomic exchanges at the In(Zn)P/ZnS interface.

The aforementioned findings from Figs 1, 2, 3, 4 are summarized in Fig. 5. The diagram shows the atomic exchanges in the NCs after 30 min and 120 min ZnS coating. For 30 min coated NCs, the ZnS shell coverage was incomplete so that the In-Zn bonds at the core-shell interface are only partially formed (Fig. 4). However, S^2−^ ions were readily incorporated into the In(Zn)P core so as to form S-In bonds (Fig. 3). After 120 min of ZnS coating the ZnS shell coverage has a saturated number of In-Zn bonds at the interface, showing passivation of the surface interaction which may be the reason for the improved photoluminescence properties of the ZnS-coated NCs. The amount of S^2−^ ions in the core was similar to that in the 30 min coated ZnS NCs. No noticeable signature of the incorporation of the core atoms into the shell was observed in the XAS spectra of the shell atoms (Figs 1 and 3).

**Figure 6:**
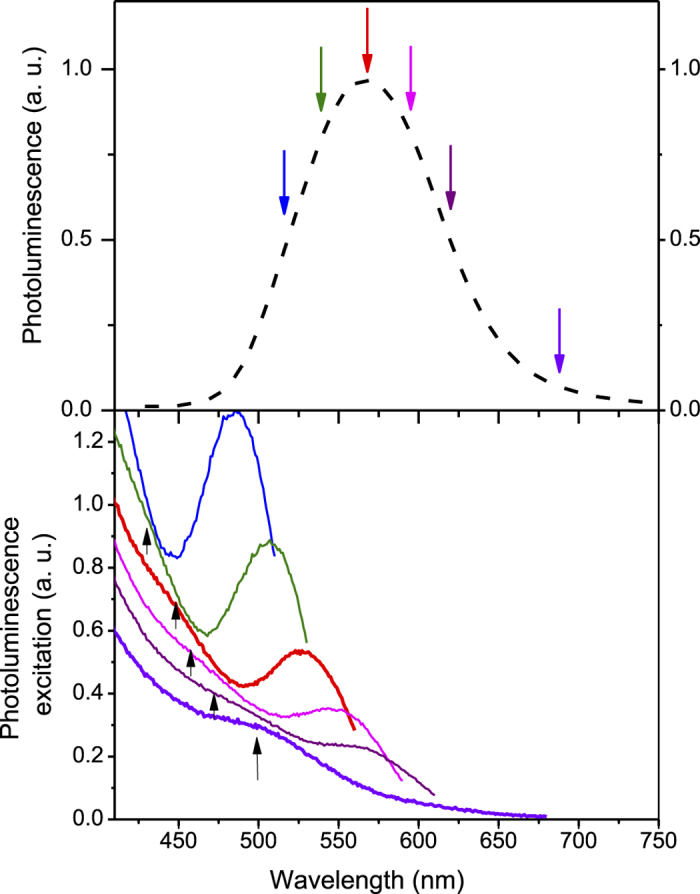
(**a**) PL and (**b**) PLE spectra using the detection wavelengths indicated by arrows in (**a**). The upward pointing black arrows show the position of the second broad peak. The vertical scales for the PLE spectra have been offset for clarity.

It is plausible that ion exchange is aided by the presence of P vacancies in the core as this would encourage the diffusion of S into the core. One indication of the presence of P vacancies is the observation of sub-bandgap photoluminescence involving surface In dangling bonds (which result from P vacancies)^13^ in photoluminescence excitation (PLE) spectra. Figure 6a shows a standard photoluminescence (PL) spectrum from the In(Zn)P cores. PLE spectra were collected by selecting a narrow range (5 nm bandwidth) of photoluminescence emission at the specific wavelengths shown by the colored arrows on the PL spectrum in Fig. 6a. The excitation wavelength was then scanned from 410 nm up to a wavelength that is 20 nm shorter than the emission wavelength and the photoluminescence at the selected emission wavelength plotted as a function of the excitation wavelength. The resulting PLE spectra from the In(Zn)P cores are shown in Fig. 6b for the six different emission wavelengths indicated by the colored arrows on Fig. 6a.

In this excitation range, one can see a sharp peak located at a wavelength which is 30–50 nm shorter than the detection wavelength as expected from the excitonic process. For example, for emission at 515 nm (blue curve in Fig. 6b), the main PLE peak is at 480 nm. In addition, for all PLE spectra except for the shortest emission wavelength (515 nm, blue curve), there is a second broad peak, whose position is indicated by the upward black arrows in Fig. 6b. This peak originates from absorption of photons across the bandgap of the nanocrystals, which generates excitons subsequently captured on the In surface states and emitted at an energy below the bandgap. For example, a broad absorption peak at 480 nm can be seen in the PLE spectrum corresponding to the emission wavelength of 620 nm (purple curve). This means that the nanocrystals which absorb light at 480 nm show excitonic emission at 515 nm and surface states emission at 620 nm. This large energy difference between exciton and surface state emission (~400 meV) is consistent with the emission from In dangling bonds introduced by P vacancies.

The selective blockage of Zn permeation into the core might ensue mostly from the abundance of Zn in the In(Zn)P core. The Zn concentration in the pristine In(Zn)P core (prior to shell growth) was roughly estimated by comparing the Zn K-edge XAS intensity with that of 30-min-coated core, in which the 3 nm-sized core is covered by a shell 1 or 2 layers thick. The estimated Zn concentration in the core was 5–10% relative to In or P, which is marginal for a dopant to maintain the structure of the InP host lattice.

On the other hand, S can easily permeate into the In(Zn)P core because there would be no such mass action as the ionic radius of S is relatively small compared to Zn and In. The concentration ratio of S_core_ to S_shell_ estimated from the intensity ratio of the main peaks in the S K-edge XAS spectrum of the 30-min-coated sample (Fig. 3) was at most 5–10%. This implies that the S concentration in the In(Zn)P core after the shell growth is comparable or smaller than the Zn concentration. Also, it was revealed in Fig. 3 that the chemical state of the S ions in the system was S^2−^ for all samples. The fact that there are no other oxidation states of S (Fig. 3) strongly suggests that sulfate (SO_4_^2−^) or sulfite (SO_3_^2−^) phases due to remnant oxygen do not form in the core. Therefore, we can conclude that the S^2−^ ions are incorporated in the core.

It has been suggested in previous studies that certain S-In-P bonds should form in the InP/ZnS core-shell structure^14^,^15^. The presence of P vacancies allow for the incorporation of S ions into the core resulting in an InP_1−x_S_x_-like complex. In this case, because S^2−^ has a lower oxidation state than P^3−^, it should act as an *n*-dopant to increase the chemical potential. In our previous study on In(Zn)P/ZnS (supporting information in ref. 10), we indeed observed that the binding energies for the core levels as measured using X-ray photoelectron spectroscopy increased after coating with the ZnS shell. The constant higher binding energy shifts indicate an increase of the chemical potential^16^ consistent with the scheme of *n*-type doping for the III-V semiconductor NCs. This evidently shows the doping effects in In(Zn)P_1−x_S_x_ NCs.

While transmission electron microscopy (TEM) is considered to be the method of choice for structural characterization of nanostructures, the dimensions and geometry of the core-shell nanocrystals studied here, combined with similar lattice constants for InP (0.5869 nm) and ZnS (0.5406 nm), make it challenging for the TEM to provide information beyond average composition and total average size. Both average compositional and size information for InP/ZnS core-shell NCs were obtained using spectroscopic (electron energy loss spectroscopy, EELS, and energy dispersive spectroscopy, EDS) and imaging (bright/dark field and high angle annular dark field, HAADF) methods. Figures S1 to S3 in the Supplementary Information show that it was impossible to distinguish the ZnS shell and InP core using TEM. While the resolution of the spectroscopic TEM techniques could be potentially improved by increasing the data acquisition time, any attempts to do so resulted in severe electron beam damage to the nanocrystals.

In this work, we have demonstrated for the first time the incorporation of shell anions into the core at the core/shell interfaces by analyzing the detailed evolution of XAS spectra. This provides insights for future studies on the concept of active interfaces in core/shell NCs. Using the high-energy resolution and very long probing depth of tender to hard XAS, we have deduced reliable chemical information on the core/shell atoms and their interactions at the interface. Our methodology could be easily extended to other core/shell structures to examine atomic exchanges. Supported by photoluminescence excitation measurements, it would be possible to construct a complete picture of what happens at nanoscale interfaces which will lead to substantial improvement in optical properties of such materials.

## Conclusion

This study has focused on elucidating the possible atomic interactions at the core-shell interface in In(Zn)P/ZnS NCs. XAS spectra from the edges of the atomic species present have revealed the selective incorporation of shell atoms into the core: the S^2−^ ions from the ZnS shell penetrate into the core whereas the Zn^2+^ ions do not. We found that S was incorporated into the core even for NCs with a thin (<1 nm) ZnS shell. Detailed analysis of the XAS peaks further demonstrated that at the early stages of ZnS deposition, the ZnS shell did not fully cover the core, while the shell coverage becomes complete gradually as growth time increases. The substantial atomic exchange at the core-shell interface necessitates a detailed model for the shell structure beyond a simple layer coverage model in order to understand the role of the reactive shells in the NCs. This may be the reason for the improved photoluminescence properties of In(Zn)P/ZnS core-shell particles as S passivates the surface of the In(Zn)P NCs due to the atomic exchange. This work demonstrates the feasibility of using a combination of XAS and PLE for a mechanistic study of other core-shell systems. The combination is especially useful in systems where it is challenging to make use of microscopic techniques to elucidate the nanostructural details. Although in the future it would be interesting to explore the use of low dose techniques in electron microscopy to study these core and thin shell systems, the simple sample preparation required for XAS and PLE makes this combination a powerful characterization method which should be useful for process monitoring in these core-shell systems.

## Materials and Methods

### Synthesis of In(Zn)P/ZnS NCs

We prepared our NCs using the single molecular precursor (SMP) method. For preparation of the In(Zn)P core, indium acetate (2 mmol), zinc stearate (1 mmol), myristic acid (7.0 mmol) and 1-octadecene (ODE, 60 mL) were mixed, heated to 100 °C and kept under a vacuum for 1 h. This solution was then purged with argon and heated up to 290 °C. Tris(trimethylsilyl)-phosphine (PTMS, 1.7 mmol) in ODE (10 mL) was prepared inside a glovebox and rapidly injected into the solution. The reaction was carried out at 290 °C for 5 min. The result was a dark red crude solution of In(Zn)P cores with a diameter of approximately 3.15 nm^10^. To form the shell, 60 mL of the crude solution of In(Zn)P was mixed with 45 mL of ODE and 10 mL of oleylamine (OLA) and stirred for 15 min. After that, 2.7 mmol of zinc dibutyldithiocarbamate (ZDBT) powder was added and stirred at room temperature for another 30 min to form the SMP-OLA complex. The solution was heated up to 170 °C and kept at this temperature for 30 min. For the 30 min sample, 10 mL of this solution was extracted, washed with an acetone-chloroform mixture (8:2 by volume) and centrifuged to separate the NCs. The estimated shell thickness from the mass ratios^10^ was equivalent to 1 or 2 layers. For the 60 min sample, an additional 5 mL of 0.355 M ZDBT-OLA was added after the 30 min sample was extracted. Likewise, another 5 mL of ZDBT-OLA was added after extraction of the 60 min sample to obtain the 120 min coated sample. Finally, the NCs were re-dispersed with nonpolar solvents onto a Si wafer and dried for the XAS measurements.

### X-ray Absorption Spectroscopy

The Zn K-edge XAS was obtained on the 8C beamline at the Pohang Light Source in Korea and the In L_3_-, S K-, and P K-edge XASs were obtained on the 16A1 beamline at the Taiwan Light Source in Taiwan. All spectra were collected using the fluorescence yield mode at room temperature.

### Photoluminescence and photoluminescence excitation spectroscopy

PL and PLE spectra were obtained using a Horiba FluoroMax© 4 spectrometer with a 15 cm integrating sphere. Monochromated Xe-lamp illumination with a 2 nm bandwidth was used for excitation and photoluminescence was measured with 2 nm resolution using a monochromator equipped with a photomultiplier tube.

## Additional Information

**How to cite this article**: Cho, D.-Y. *et al.* The role of ion exchange in the passivation of In(Zn)P nanocrystals with ZnS. *Sci. Rep.* **6**, 22818; doi: 10.1038/srep22818 (2016).

## Supplementary Material

Supplementary Information
