# Supplementary material for: The role of ion exchange in the passivation of In(Zn)P nanocrystals with ZnS
Source: Sci Rep. 2016 Mar 14;6:22818. doi: 10.1038/srep22818 (PMC4789599; doi:10.1038/srep22818)
Supplement: Supplementary Information [file srep22818-s1.doc]

**Supplementary Information**

The role of ion exchange in the passivation of In(Zn)P nanocrystals with ZnS

Deok-Yong Cho^1^, Lifei Xi^2^, Chris Boothroyd^3^, Beata Kardynal^4^, Yeng Ming Lam^5^

^1^IPIT & Department of Physics, Chonbuk National University, Jeonju 561-756, Republic of Korea

^2^Institute of Solar Fuels (EE-IF), Helmholtz Zentrum Berlin (HZB), 12489 Berlin, Germany

^3^Ernst Ruska Centre (ER-C), PGI-5, Forschungszentrum Jülich, 52425 Jülich, Germany

^4^ Semiconductor Nanoelectronics (PGI-9), Forschungszentrum Jülich, 52425 Jülich, Germany

^5^School of Materials Science and Engineering, Nanyang Technological University, 639798, Singapore


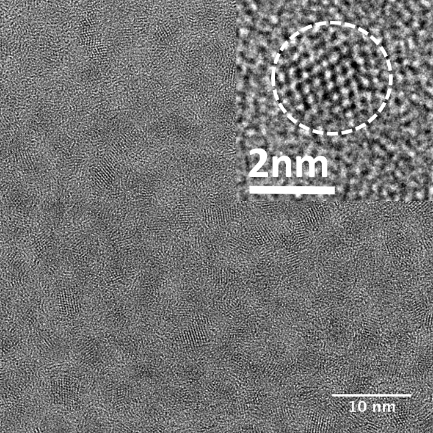


(a)


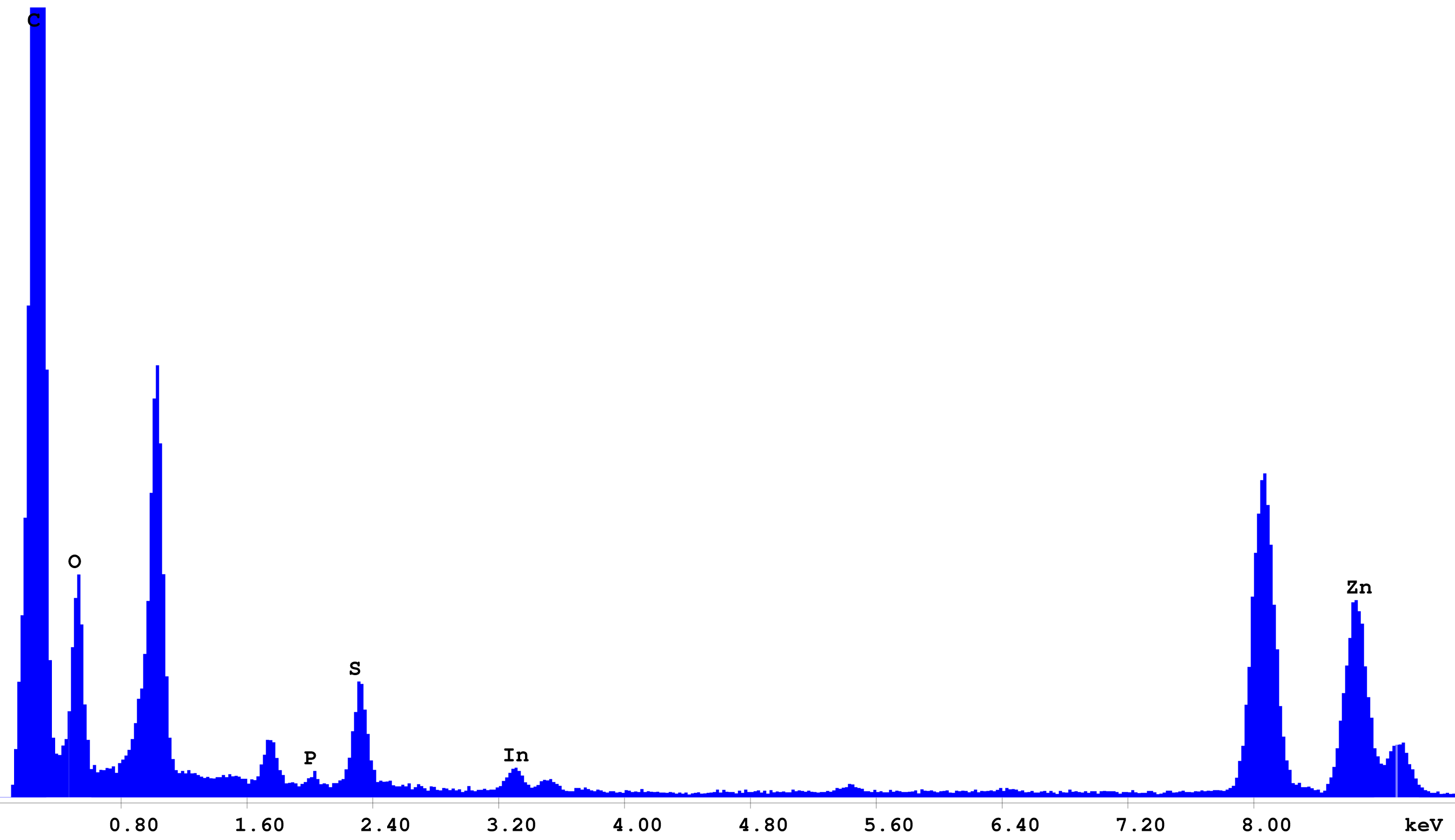


(b)

**Figure S1.** (a) HRTEM image of In(Zn)P/ZnS NCs synthesized with ZDBT after 120 min of coating at 170 °C. Inset: Enlargement of one In(Zn)P/ZnS NC. (b) Energy-dispersive X-ray spectrum from the In(Zn)P/ZnS NCs shown in (a).


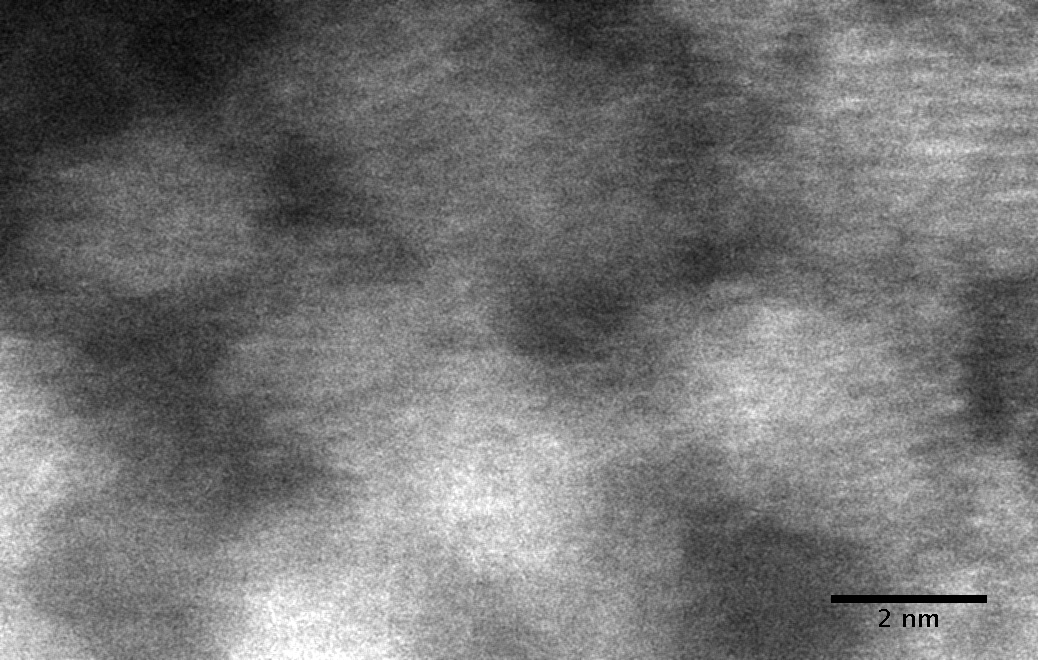


(a)


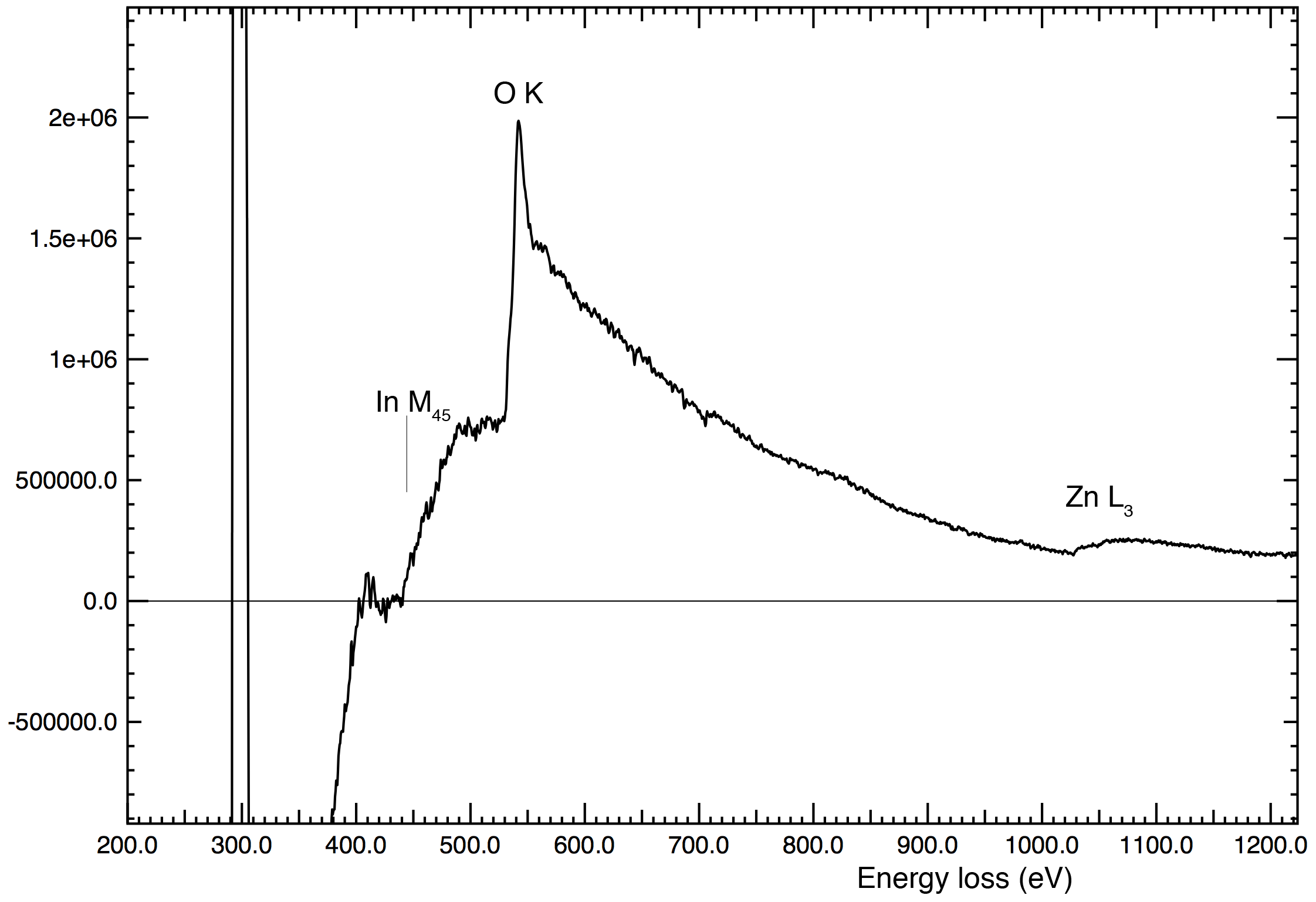


(b)


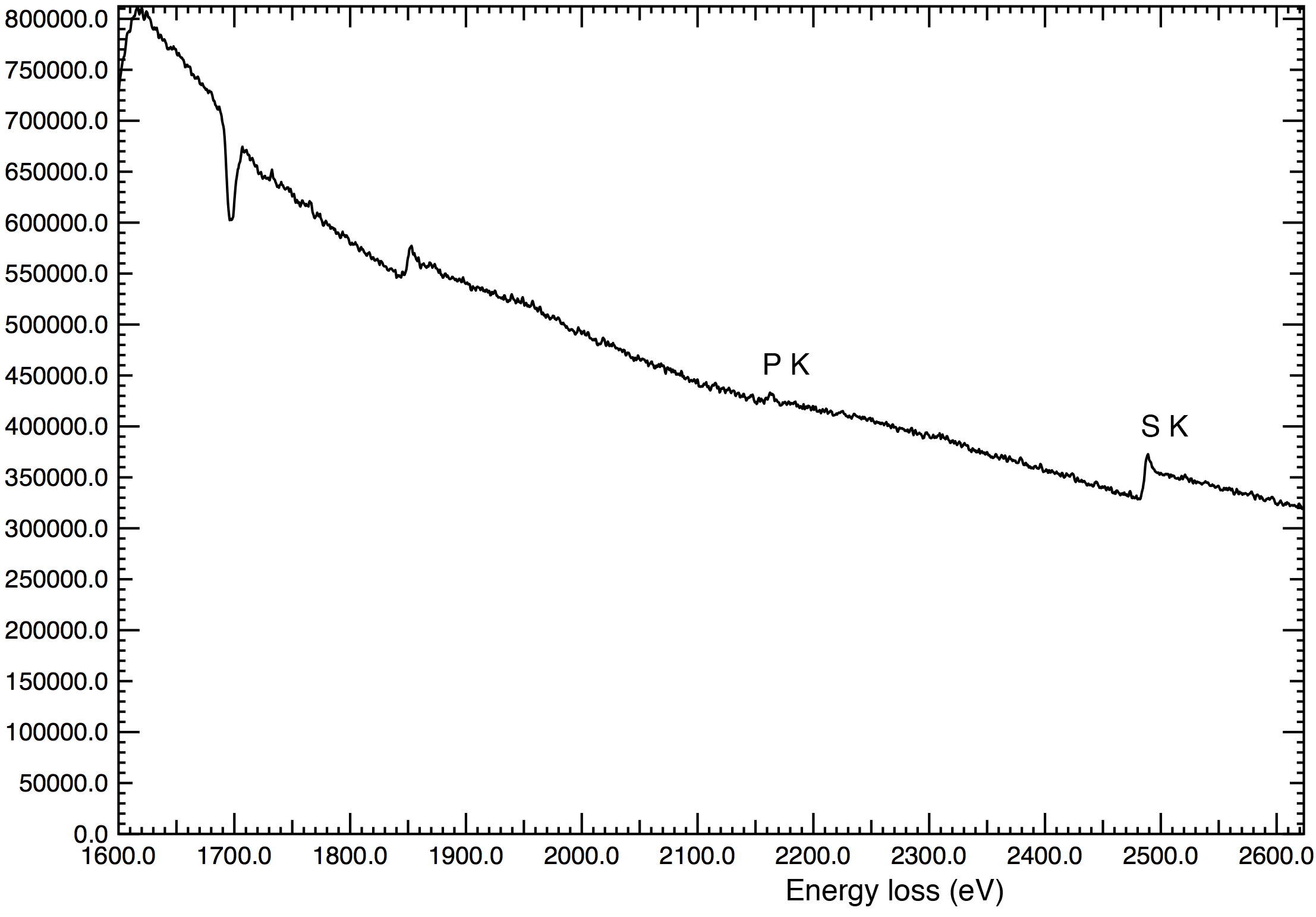


(c)

**Figure S2.** (a) HAADF STEM image of the In(Zn)P/ZnS NCs. (b) Background subtracted EEL spectra for energy losses from 200 to 1200eV showing the Mo M_45_, O K and Zn L edges. The feature at 300eV is part of the C K edge. (c) EEL spectra for energy losses from 1600 to 2600eV showing the P K and S K edges. The edge at 1839eV is the Si K edge from the carbon support film and the dip at 1700eV is an artifact resulting from earlier over-exposure of the CCD detector by the zero-loss peak.


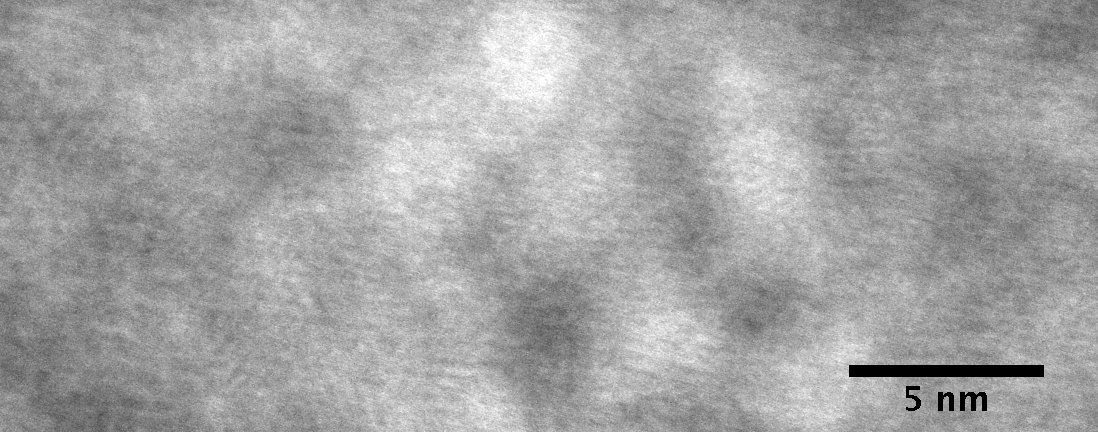


(a)


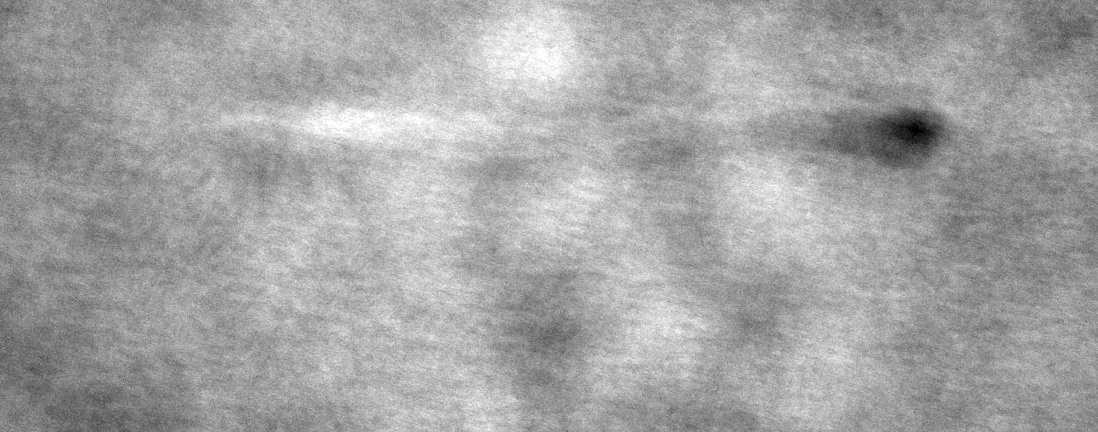


(b)

(c)


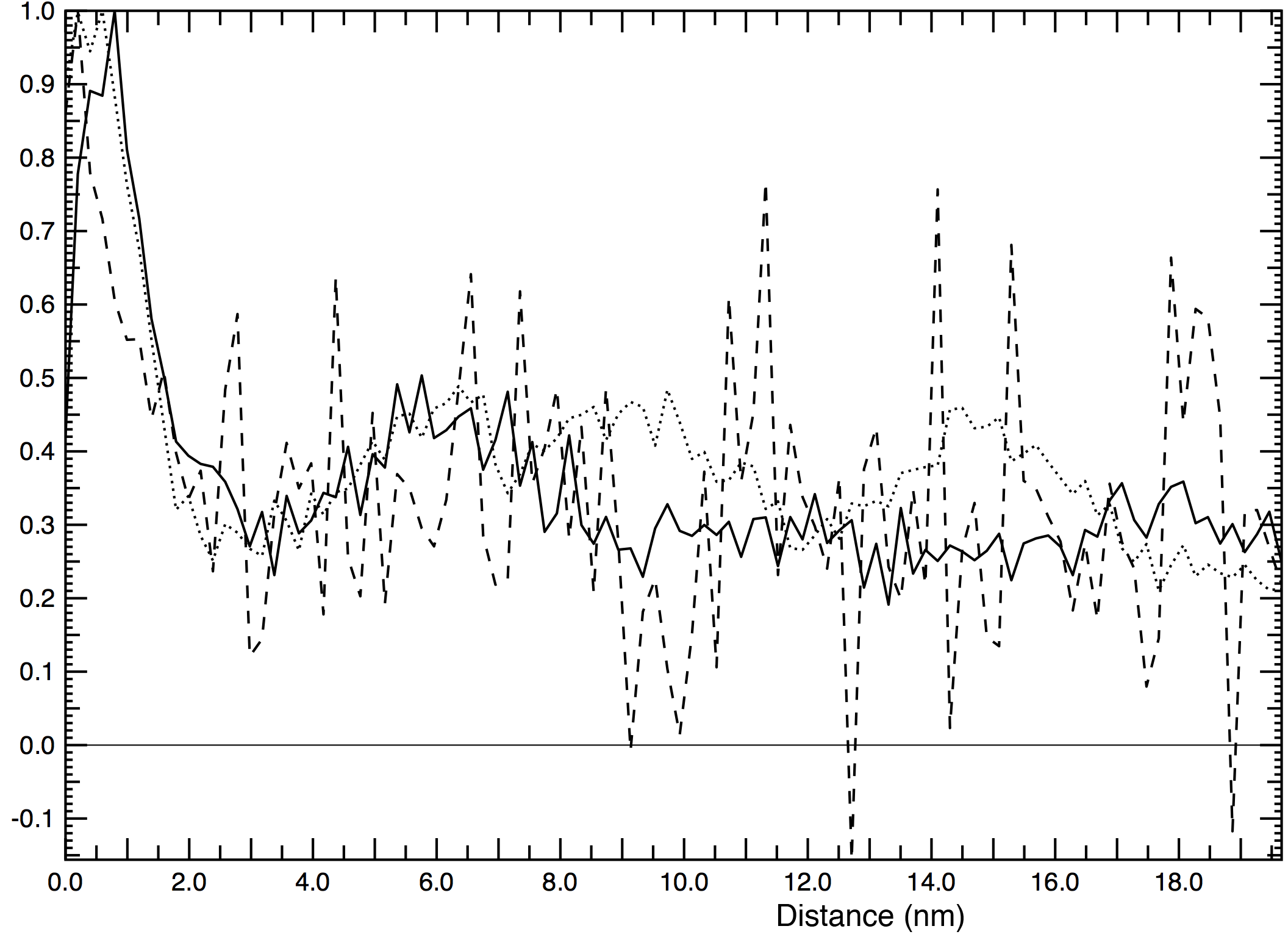


**Figure S3.** HAADF STEM images from the In(Zn)P/ZnS NCs (a) before and (b) after the EELS linescan shown in (c). The position of the line scan can be seen from the electron beam damage visible in (b). (c) Linescan of EELS edge intensities across several In(Zn)P/ZnS NCs. The vertical scale represents the relative areas of the P L (solid line), S K (dotted line) and In M (dashed line) edges. The edge areas decrease after the first nm due to electron beam damage.
